# Post-Transcriptional Control in the Regulation of Polyhydroxyalkanoates Synthesis

**DOI:** 10.3390/life11080853

**Published:** 2021-08-20

**Authors:** Alexandra Peregrina, João Martins-Lourenço, Filomena Freitas, Maria A. M. Reis, Cecília M. Arraiano

**Affiliations:** 1 Control of Gene Expression Lab., Instituto de Tecnologia Química e Biológica António Xavier, Universidade Nova de Lisboa, 2780-157 Oeiras, Portugal; joaopm@itqb.unl.pt; 2 Associate Laboratory i4HB-Institute for Health and Bioeconomy, School of Science and Technology, NOVA University Lisbon, 2829-516 Caparica, Portugal; a4406@fct.unl.pt (F.F.); amr@fct.unl.pt (M.A.M.R.); 3 UCIBIO—Applied Molecular Biosciences Unit, Department of Chemistry, School of Science and Technology, NOVA University Lisbon, 2829-516 Caparica, Portugal

**Keywords:** polyhydroxyalkanoates, mcl-PHA, scl-PHA, post-transcriptional regulation, riboregulation, small non-coding RNAs

## Abstract

The large production of non-degradable petrol-based plastics has become a major global issue due to its environmental pollution. Biopolymers produced by microorganisms such as polyhydroxyalkanoates (PHAs) are gaining potential as a sustainable alternative, but the high cost associated with their industrial production has been a limiting factor. Post-transcriptional regulation is a key step to control gene expression in changing environments and has been reported to play a major role in numerous cellular processes. However, limited reports are available concerning the regulation of PHA accumulation in bacteria, and many essential regulatory factors still need to be identified. Here, we review studies where the synthesis of PHA has been reported to be regulated at the post-transcriptional level, and we analyze the RNA-mediated networks involved. Finally, we discuss the forthcoming research on riboregulation, synthetic, and metabolic engineering which could lead to improved strategies for PHAs synthesis in industrial production, thereby reducing the costs currently associated with this procedure.

## 1. Introduction

### 1.1. The Age of Plastics

Petroleum-based plastics are pervasively used and appear as cheap and easy to make but at the cost of the environmental toll [1,2]. Eight million tons of plastic end up in the oceans every year, where they break down into micro-and nanoplastics [3,4]. Plastics have also been found falling out of the air in several mountain locations. This discovery suggests that, after the evaporation of the water, microplastics are carried around the planet in atmospheric winds, becoming part of the breathable air [5]. The impact of deposition of waste plastics in the land is also extremely relevant. Animals eat plastic and can get wrapped up, trapped, or asphyxiated by them [6]. In addition, plastics can easily enter the food chain and have adverse consequences for humans. During their processing and consumption, they release toxic additives that were used to shape them, harden them, or make them flexible, and these additives can enter into the food chain and water supply. For instance, bisphenol A (BPA), a common precursor of widely used plastics, was found in the urine of approx. 93% of the 2517 individuals tested in a study [7]. In addition, these molecules could interfere with our endocrine system since they are thought to adopt hormonal functions in the human body [8]. 

Therefore, to break the plastic wave, bio-based and biodegradable alternatives to synthetic plastics should be considered [9,10], especially with the drastic increase in plastic pollution due to the current COVID-19 pandemic [11,12]. However, the elevated cost of industrial procedures and lack of significant large-scale production [13,14,15,16], together with the availability of appropriated carbon sources, have limited faster progress in these processes and consequently greater market penetration [17,18].

### 1.2. Polyhydroxyalkanoates: Bio-Based Biodegradable Plastics

**Figure 1:**
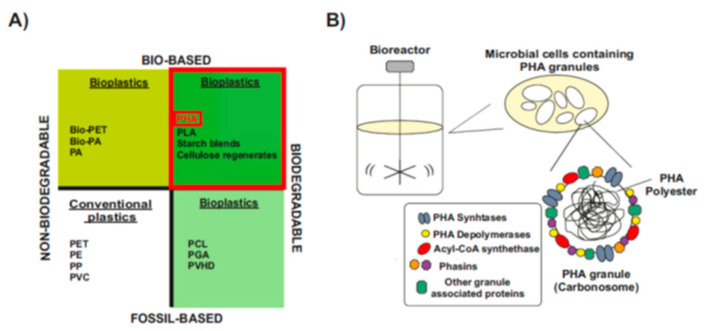
Material coordinate system of plastics. (**A**) Type of plastics. Division of plastics into four groups, according to their biodegradability and biological origin. Upper-right, PHA: polyhydroxyalkanoates-are biodegradable polymers naturally produced by numerous microorganisms (Modified after [16,19]). **(B**) PHAs: bio-based biodegradable plastics. When a carbon substrate is present in excess, in parallel to depletion of other nutrients essential for biomass formation, PHAs are stored in the form of cytoplasmic spherical inclusions. These PHA granules are multi-complexes usually called “carbonosomes”. They contain a hydrophobic core surrounded by PHA granule-associated proteins, such as PHA synthase, PHA depolymerases, regulatory and structural proteins (Modified after [24,29]).

The word “bioplastics” has commonly been used to make a distinction from petrochemical polymers, which is partially misleading, since not all types of bioplastics are bio-based and biodegradable [16,19] (Figure 1A). Some bioplastics are biodegradable but fully fossil-based. Their chemical structure can be degraded in a slow process catalyzed by enzymes of some aerobic and anaerobic microorganisms that are widely distributed in various ecosystems. However, they are not biodegradable in animal bodies and sometimes they remain in marine waters [16,20] (Figure 1A, bottom-right). Others are bio-based but chemically identical to their fossil counterparts, so they are not biodegradable [16,21] (Figure 1A, upper-left).

Only bio-based and biodegradable bioplastics are more ecologically friendly and serve as the best substitute for conventional plastics (Figure 1A, upper-right). Among them, one of the most promising class of bioplastics are the bacterial polyesters polyhydroxyalkanoates (PHAs), which are produced through industrial bacterial fermentation of sugar or lipids by numerous Gram-positive and Gram-negative bacteria [16,20]. Inside the cells, PHAs molecules aggregate to form water-insoluble granules, the carbonosomes, which are intracellular reserves of energy during starvation [22,23] (Figure 1B). In carbonosomes there is a constant cycle of synthesis and degradation, and this bidirectional process is a great advantage in the adaptation to rapid changes in the environment [24,25]. During the last few years, PHAs are being proclaimed as the best alternative to fossil-based plastic due to their good balance between biodegradability rate, material properties that range from thermoplastics to elastomers, and the possibility to be processed into different final products [9,10,26]. However, production costs of PHAs are still too high when compared to the synthetic plastics [13,14]. Although they have not yet reached industrial scale, in the last decade a more cost-effective processes for the production of PHA have been developed based on the use of wastes, industrial products and less energy-demanding approaches [27,28]. Once the process scale constraints are overcome, PHA will become more competitive and replace the synthetic plastics in many applications. 

### 1.3. Types and Chemical Structure of PHAs Polymers

**Figure 2:**
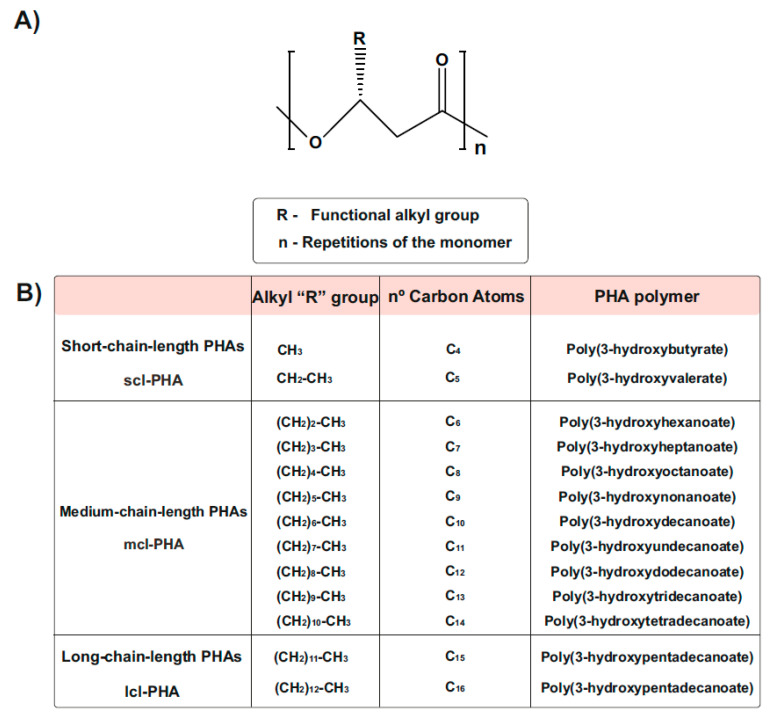
Chemical Structure of polyhydroxyalkanoates (PHAs). (**A**) General structure of a PHA molecule. The ’R’ functional group represents the alkyl side chain, and the number of repetitions of the monomeric unit is given by ‘n’. (**B**) PHAs can be classified as scl-PHAs, mcl-PHAs, and lcl-PHAs, depending on the carbon numbers in the monomeric constituents (modified after [31]).

PHA generally consists of (R)-hydroxy fatty acid monomer units, which contain an alkyl side chain R group that varies in carbon length from methyl (C1) [30,31] (Figure 2). These polymers are usually divided into three different types, according to the number of carbons in the monomeric subunits [31]. Short-chain-length PHA (scl-PHA) polymers are composed of monomers containing 3 to 5 carbon atoms, whereas medium-chain-length PHA (mcl-PHA) polymers are composed of monomers containing 6 to 14 carbon atoms. The third type are the long-chain-length PHAs (lcl-PHA), with a minimum 15 carbons [30,31] (Figure 2B). Their chemical properties are different and depend on the bacterial host and the fermentation conditions used for their production, making them suitable for different purposes. Scl-PHAs are highly crystalline, which makes them relatively stiff and brittle [30,32]. However, polymers with a greater number of carbons are more flexible and elastic, resulting in increased research interests [33].

PHAs are classified into homopolyesters, with only one variety of monomer, and heteropolyesters, which can be subdivided into copolyesters (monomers differing in either backbones or side chains) and terpolyesters (different side chains and backbones) [29,34]. The so-called polyhydroxybutyrate (PHB) is one of the most common homopolymer PHA and best studied scl-PHA, containing the shortest possible side chain with only one methyl group [35,36] (Figure 2B). The mechanical properties of PHB are comparable to conventional fossil-based plastics such as polypropylene or polyethylene [30,32], and are reaching new interest for applications in medicine, where chemical composition and product purity are crucial [37]. Other uses are food packaging and containers, utensils, biofuel, bottles, and disposable personal hygiene [30,32,38]. mcl and lcl-PHAs can be produced from many different substrates and have been studied in numerous bacterial species, particularly in pseudomonads [27,39]. The versatility in the physical properties of mcl-PHAs makes those materials appropriate for a wide range of applications, including daily use and medical purposes [10,28]. As described in [26,38], the uses comprise tissue engineering, orthopedic, urological and cardiovascular devices, wound management, and drug delivery, among others.

### 1.4. Natural PHA Producers and Engineering of Non-PHA Producers

Although the list of natural PHA producers is large and includes extremophile bacteria, mainly Gram-negative species have been explored for their capacity to synthesize PHAs. Among this list, the most known are: *Cupriavidus necator* (previously *Ralstonia eutropha*), *Azotobacter vinelandii*, and *Burkholderia* spp., as scl-PHA producers [31,40,41]; *Pseudomonas* strains (especially *Pseudomonas putida*), as mainly mcl-PHA accumulators, while some strains are able to produce scl-mcl-PHA co-polymers [15,33,42].

Natural PHA-producing bacteria usually harbor the enzymatic repertoire for polymer degradation and are often difficult to lyse, which makes the recovery of PHA laborious and expensive. For this reason, engineered bacteria are currently being utilized as an alternative in the industrial PHA production, carrying *pha* biosynthetic genes, with *Escherichia coli* as one of the most used hosts [15,43,44]. 

In the last few years, new knowledge was gained about biosynthetic pathways (largely confined to Acetyl-CoA precursors) and the enzymes involved in PHAs accumulation [35,45]. Nevertheless, several aspects still remain elusive, and it is quite important to be able to regulate and improve the process.

### 1.5. PHA Composition and Preferred Carbon Source

To produce PHAs, bacteria can use different carbon sources as substrate such as saccharides, fatty acids, alcohols, or gases [31,46]. Generally, different bacteria have preference for one of them depending on the metabolic pathways they harbor, so the metabolic routes in which those substrates are integrated are different, as well as the final product composition [31,35,47]. The metabolic flux from the intermediary acetyl-CoA to different PHA compositions is greatly dependent on nutrient conditions and the supplied carbon source [31,47]. Under carbon-rich conditions, the level of cellular coenzyme A increases substantially, causing the oxidation of acetyl-CoA into the Krebs cycle for energy production and cell growth. However, in the presence of unbalanced C/N conditions, acetyl-CoA can be used for the PHA synthetic pathways [31,47,48].

The genes (*pha*) that regulate the synthesis and degradation of PHA at the transcriptional level are widely known among the prokaryotes. In the extensively studied *P. putida,* the genetic organization of the *pha* genes integrates a very conserved *pha* cluster composed by two synthases (*phaC1* and *phaC2*) responsible for the PHA synthesis; a depolymerase (*phaZ*) encoding for the PHA mobilization; the transcriptional regulator (*phaD)*; and the regulatory and functional phasins (*phaF* and *phaI*) [12,25,49]. In the last few years, new knowledge has been deciphered about the PHAs synthesis and degradation in pseudomonads and other organisms [33,50,51,52]. However, the molecular regulation at the post-transcriptional level of PHA synthesis is still unclear and needs further investigation.

### 1.6. RNA World

Post-transcriptional control of gene expression involves important enzymes such as ribonucleases (RNases), and bacterial small non-coding RNAs (sRNAs) [53,54,55,56,57]. In recent decades, RNA regulators were shown to be a key step in the control of many cellular processes. sRNAs are not translated into proteins and have the ability to post-transcriptionally modulate and regulate gene expression, in response to specific environmental or physiological signals, facilitating adaptation to diverse environmental stresses [58,59,60]. 

As reported for many other cellular processes, riboregulation has also been involved in the production of PHAs in different bacterial organisms. Herein, we describe the published work on different bacteria, where post-transcriptional control is the protagonist during the bioplastics synthesis; either shown to be involved in the control of important genes or used as a tool to control them [61,62,63,64].

This review enables the reader to acquire better knowledge on the molecular mechanisms underlying the bacterial accumulation of biopolyesters, emphasizing the post-transcriptional control, a neglected cellular regulation mechanism, as indicated by the reduced bibliography that is available. Furthermore, we provide new insights for the future domestication of microorganisms, which, in our view, have the potential to improve quality and reduce costs in industrial production of PHAs.

## 2. RNA-Mediated Control in Native Synthesis of PHAs

### 2.1. The Expanding RNA World: Non-Coding Bacterial RNome

For years, it was considered that the expression of the bacterial genome resulted in three large groups of RNA molecules: mRNA, which contains open reading frames that translate into proteins, and two more types of RNA, the ribosomal rRNA and transfer tRNA, which are essential for protein biosynthesis carried out by ribosomes. Therefore, regulation of gene expression was exclusively associated with the activity of protein regulators [65,66]. However, post-genomic research is revealing an unprecedented high abundance and diversity of untranslated small RNA molecules (50–350 nt of average length) called sRNAs or non-coding RNAs, expanding the total of RNA species that together constitute the bacterial “RNome” [60,67]. These sRNAs are commonly encoded by single transcriptional units between open reading frames (ORFs) and although do not translate to protein, play very important roles in the gene regulation of diverse physiological processes at the post-transcriptional level [58,60,68].

These riboregulators are deeply conserved in prokaryotes and adjust gene expression in response to specific environmental or physiological signals, facilitating adaptation to diverse environmental stimuli. This is especially important to allow the cell to profit from transiently available nutrients [60,69]. As with other RNA molecules, the sequence and structure of sRNAs determine their function [70]. Their activity depends on their cellular abundance, regulated by the balance between their transcription and degradation rates [71,72].

Depending on their genomic location relative to the mRNA targets that they regulate, sRNAs are classified as cis- or trans-encoded. The latter constitute a majority group and are expressed from intergenic regions (IGRs), generally far from the target messenger counterparts [59,73,74]. These riboregulators act on the activity of specific genes. It can be directly binding to mRNAs or through the control of post-transcriptional regulatory proteins by mimicking their mRNA substrate [60,68,75], e.g., the well-characterized CsrB/CsrC family of *E. coli* sRNAs [76]. However, most of these riboregulators involves their hybridization with an imperfect, short, and discontinuous series of complementary nucleotides (at least 6–7 base pairs) which are usually located in the region of translation initiation (5′-UTR) of its trans-encoded target messengers [59,73]. 

The hybridizations between sRNA-mRNA are generally facilitated by RNA chaperones such as Hfq or FinO/ProQ, thus directly competing with ribosome access [77,78]. sRNAs typically act at the level of mRNA stability and/or translation efficiency which usually results in translation blockage and subsequent degradation of mRNA by cellular ribonucleases [60].

#### 2.1.1. RNA-Binding Proteins and Regulatory Networks

Besides sRNAs, another essential type of prokaryotic post-transcriptional regulators is the RNA-binding proteins (RBPs). In recent years, more proteins were identified, and new insights were gained into their diverse mechanisms of action to regulate their own activity and the expression of their target genes in different bacteria [79]. This mode of action is reviewed by [80,81].

Some regulatory RBPs can act as chaperones by facilitating the intermolecular base pairing between sRNAs and mRNAs [80,82]. One of the best characterized RBP is Hfq that also exerts a central role in post-transcriptional gene regulation, as evidenced by the pleiotropic effect of the inactivation of the *hfq* gene in many Gram-negative bacteria [53,56,83,84].

The Hfq protein was discovered almost 50 years ago in *E. coli* as a host factor required for the replication of the RNA phage Qβ [85]. Structurally, it is a homo-hexameric ring that exposes two different positively charged surfaces (proximal and distal faces), which constitute alternative binding sites that can discriminate between RNA molecules, with the proximal face being important for the binding of U-rich sequences [86,87]. As most sRNAs have typical bacterial Rho-independent terminators that usually contain a poly-U 3′-terminus, Hfq can interact with the terminators and influence sRNA stability [77]. The distal face has strong affinity for A-rich sequences of mRNAs [87,88]. The rim of the Hfq ring has been reported to be required for interacting with some sRNAs, comprising a third interaction site [87,89]. Hence, each Hfq ring is able to simultaneously bind different RNA molecules or even a single molecule, bridging both faces around the oligomer rim. If a sRNA binds on one face and a cognate target mRNA does so on the second face, this ternary complex will lead to productive RNA duplex formation [89]. Hfq has also been shown to be important for ribosome biogenesis and affects translation fidelity [90].

Hfq also offers a scaffold for the interaction with several other proteins [84], e.g., Crc, which is involved in catabolite repression control in some *Pseudomonas* sp. [80,84,91,92]. This protein has been involved in the post-transcriptional regulation of polyhydroxyalkanoates synthesis in *Pseudomonas putida*, together with several sRNAs [61,91,93,94,95,96]. More details will be described in Section 2.2. There are other prokaryotes where sRNAs seems to be implicated in the accumulation of different types of PHAs, but the action of Hfq in this process remains elusive [62,64]. In the last few years, new knowledge was gained about the repercussion of the regulatory networks of RNA-binding proteins in PHAs accumulation. Nevertheless, numerous aspects still remain unclear and need further investigation. In Section 2.2, the identified relation of these proteins with the synthesis of PHAs in some organisms is described in more detail.

#### 2.1.2. Post-Transcriptional Regulation by Ribonucleases

Ribonucleases are enzymes that have been widely described to play important and even essential roles. As reviewed in [53,72,97], RNases are essential participants during the post-transcriptional regulation and as key modulators of RNA decay. In general, sRNAs are mainly degraded by RNase E and PNPase, or by RNase III, if the sRNA is hybridized to an mRNA target [53]. In this review, we mainly focus in the model *E. coli* and other closely related organisms.

RNase E is the major bacterial endoribonuclease and cleaves single-stranded regions of structured RNAs with preference for 5′-ends and AU-rich sequences [98]. This ribonuclease is the main enzyme forming the degradosome, a ribonucleoprotein (RNP) complex involved in the decay of many RNAs [97,99]. RNase E, together with Hfq-sRNA RNP, leads to translational repression and rapid target mRNA degradation. However, Hfq binding itself (in the absence of RNase E and RNA-RNA interaction), is sufficient to mediate translational repression, destabilization, and degradation of the target mRNA [99,100]. Furthermore, in these RNP complexes, Hfq is able to protect several RNAs from cleavage by RNase E [53,101]. Recently, it was shown that Hfq can also complex with the exoribonuclease PNPase, facilitating bacterial riboregulation [102].

Another RNase involved in post-transcriptional regulation by bacterial sRNAs and through the decay of some mRNAs is the double-strand specific RNase III [103,104]. It is a highly conserved enzyme specific for double-stranded RNAs which shows preference for continuous RNA duplexes of 20–40 bps. Perfect antisense/sense RNA duplexes formed in sRNA–mRNA interactions constitute an optimal substrate for this enzyme [105].

YbeY is an additional RNase that has been recently proposed to be required for the sRNA-mediated post-transcriptional silencing of prokaryotic genes. This endoribonuclease cleaves double-stranded RNA and could have catalytic and/or Hfq-like protective functions essential for RNA metabolism and small RNA (sRNA)-mediated regulation [54].

Although the knowledge about ribonucleases is continually increasing, nothing has been published about their influence on cellular PHAs accumulation. Nevertheless, the reported relevance that RNases have for the cellular efficiency [54] suggests that they also could be a major player in the post-transcriptional control of biopolymers synthesis.

### 2.2. Post-Transcriptional Regulation of sRNAs and Their Implications for Microbial PHAs Synthesis in Different Microorganisms

Despite the importance of the post-transcriptional regulation in natural and synthetic systems, its involvement in the control of PHA synthesis continues to be a forgotten cellular regulation mechanism. This fact is exemplified by the limited number of research articles published to date. Accordingly, in one of the best studied natural producer of PHB (*Cupriavidus necator,* formerly called *Ralstonia eutropha*) [41,106], the role of riboregulation in this process remains undeciphered. Likewise, the production of PHAs in recombinant *E. coli* has been widely reported [38,43,44]. However, post-transcriptional control has not been described for the polymer synthesis in this organism. 

Subsequently, below, we review the published works that define riboregulation as a major player for the bacterial accumulation of PHAs.

#### 2.2.1. MmgR sRNA Is a Negative Regulator of PHB Accumulation in *Sinorhizobium meliloti*

*Sinorhizobium meliloti* is a natural PHAs producer in nature. This nitrogen-fixing alphaproteobacterium is able to undergo symbiosis with leguminous host plants from the genera *Medicago*, *Trigonella*, and *Melilotus* [107]. During the free-living phase, *S. meliloti* can synthetize PHB, the shortest polymer, along with other PHAs and its main carbon and reducing power storage under C/N overbalance [64,108]. However, the role of the storage of PHB during the symbiosis is yet to be determined [64,109].

The *S. meliloti* genome is distributed in three replicons (3.65-Mb chromosome, the megaplasmids 1.35-Mb pSymA, and 1.68-Mb pSymB) and encodes more than 500 sRNA candidates [56,110]. However, with only a few exceptions, the regulatory targets and mechanism of action of this repertoire of sRNAs are still unknown [55,64,111,112]. The *S. meliloti* trans-encoded sRNA, MmgR (standing for Makes more granules Regulator) is an Hfq-dependent sRNA [56,113], transcribed from the chromosome as a 77-nt RNA [64,114,115]. It is highly conserved in α-proteobacteria, as a member of the αr8 RNA family, and has been explored for its regulatory function only in *S. meliloti* [64,115]. Lagares et al. [64] found that MmgR is a negative regulator of PHB accumulation since the deletion of an internal conserved core of the sRNA gene resulted in larger cells containing 20% higher amounts of PHB (Figure 5 in ref. [64]). Further, the *mmgR* expression was described to be modulated by the availability of N existing in the growth medium [64,116].

Phasins mediate stabilization of the granule–cytoplasm interphase [117]. In agreement with this, quantitative reverse transcription-PCR (qRT-PCR) and proteomic profiling enhanced the accumulation of PhaP1 and PhaP2 proteins in the *mmgR^Δ33–51^* mutant without affecting their mRNAs levels. These decoupling results evidenced the post-transcriptional negative regulation that the MmgR sRNA carries out, in a direct or indirect manner, on the *phaP1* and *phaP2* mRNAs in *S. meliloti* [64].

The promoter activity of *mmgR* is controlled by the quality and/or amount of the available N source, reaching the highest intracellular level with nitrate as the N source or upon starvation of the organic N sources [64,118]. The expression of MmgR was mainly regulated at the transcriptional level by at least the N and C metabolism master regulators NtrC and AniA, respectively. This regulation relies on a conserved dyadic motif located within the −35 and −10 boxes of the *mmgR* promoter, and results in positive control of gene expression by the C:N molar ratio in the growth medium, upon N depletion. On the other hand, the global carbon flux regulator, AniA (PhaR), negatively controls the sRNA expression, assuming a consistent negative feedback loop on phasin and *phaZ* genes since the MmgR sRNA down-regulates PhaP1 and PhaP2 protein levels [64,116].

#### 2.2.2. Post-Transcriptional Control of PhbR as Key Step during PHB Production in *Azotobacter vinelandii*

*Azotobacter vinelandii* is a widely distributed gram-negative bacterium and a member of the family *Pseudomonadaceae* [119,120]. Species belonging to the *Azotobacter* genus are aerobic diazotroph organisms that dwell in soils worldwide, and are relevant to the development of sustainable agriculture [121,122]. This organism is a well-known model with large potential for biotechnological applications in the industry sector, due to its ability to grow on an extensive variety of substrates to produce PHBs [123,124]. The versatility of this bacterium in using low-cost unrefined carbon substrates can make the process economically competitive, making it a more sustainable bioplastic alternative [124,125]. 

The main regulatory mechanism leading to the accumulation of PHB in this organism involves the *phbBAC* operon, which encodes for key enzymes of the PHB biosynthesis pathway. This operon is in turn controlled by the transcriptional activator PhbR and the sigma factor RpoS [126,127]. Interestingly, PhbR expression has been reported to be post-transcriptionally controlled by the two-component GacS–GacA global regulator [128]. This system (global antibiotic and cyanide control) belongs to the Gac–Rsm cascade and is involved in the regulation of many cellular processes in numerous bacterial organisms, as reviewed by Lapouge et al. [129]. 

Hernandez-Eligio et al. stated that the Gac–Rsm signal transduction regulates PHB synthesis in *A. vinelandii* UW136, where GacA and RsmA acquire opposite roles, acting as positive and negative regulators, respectively. In consequence, the inactivation of *rsmA* resulted in increased PHB production, compared to the UW136 wild-type strain, and opposed to the *gacA* mutation, where PHB synthesis was scarce [128]. As described in other bacteria, GacA is expected to activate the transcription of CsrB/RsmZ/Y/X small RNAs, which, in counteracting activity with the CsrA/RsmA proteins, post-transcriptionally regulates their mRNA targets [126,129,130]. Accordingly, GacA was required for the expression of one RsmY and seven RsmZ sRNAs existing in *A. vinelandii* UW136 which interact with RsmA, and highly conserved binding sequences of the GacA were also found in these sRNAs genes [128,130,131]. Hernandez-Eligio et al. [128], moreover, revealed that the RsmA protein targets and causes instability on both, *phbR* and *phbB* mRNAs. Further analysis uncovered that the mutation in the *rsmA* gene generates an increase in the translation of *phbR/phbB*, whereas a strong reduction in their activity was observed in the *gacA* mutant, without determining whether the mutation affects the translation of *phbB*. Taken together, these results confirmed that the Gac–Rsm system controls *phbR* expression at the post-transcriptional level in this strain, while it could not be established for the regulation on *phbB* [128,130]. The model shown in Figure 7 of Hernandez-Eligio et al. [128] properly summarizes this regulatory control of PhbR by the Gac–Rsm cascade. It is also possible to consider RsmA (CsrA) as the central component of the system. Therefore, additional research (on the *phbB* regulation and the interaction of the sRNAs with the RsmA protein) is needed for the further understanding of the control of PHB through this regulatory cascade in *A. vinelandii* UW136 [128,132].

**Figure 3:**
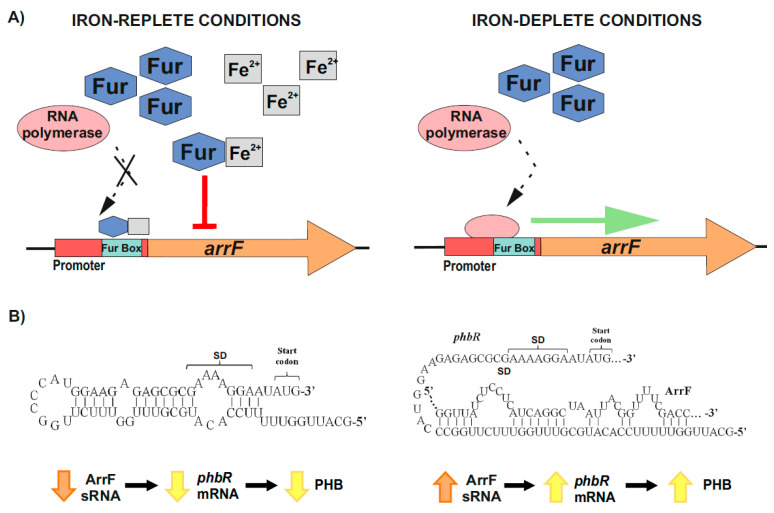
Iron-mediated regulation of PHB accumulation through the ArrF sRNA in *A. vinelandii*. (**A**) Upper left panel, under iron-replete conditions, Fur–Fe^2^^+^ complexes are established and repress *arrF* genes transcription by binding the Fur boxes in their promoters; Upper right panel, under iron-deplete conditions, RNA polymerase accesses the *arrF* promoter with its subsequent transcription. (**B**) The mechanism through which ArrF sRNA is involved in the PHB accumulation differs between different genetic backgrounds. Left panel, in *A. vinelandii* strain UW136, a predicted occluding hairpin around the *phbR* 5′-UTR could prevent its translation; Right panel, the targeting of *phbR* 5′-UTR by the ArrF sRNA could make the RBS available for translation, resulting in turn in an increase in PHB accumulation (modified after [63]).

On the other hand, and over the past few years, it has been revealed that bacterial iron-regulated sRNAs have important modulating roles (e.g., in iron homeostasis) according to the levels of this essential and potentially toxic micronutrient [63,133,134]. The genes that encode for these small RNAs hold in their promoter regions the conserved Fur or iron boxes, which function as binding sites of the ferric uptake repressor (Fur) [130,133,134,135,136]. Under iron-replete conditions, a Fur–Fe^2+^ complex is formed, which binds to the iron boxes of the sRNAs-coding genes involved in iron homeostasis, and represses their transcription. On the contrary, when iron is scarce, RNA polymerase is able to access the promoters of these genes, resulting in their transcription [130,133,134,135,136] (Figure 3A). In *A. vinelandii,* iron regulates the accumulation of PHB through one of these sRNAs, ArrF, while the mechanism seems to vary between different genetic backgrounds [63,124,130,137]. Muriel-Millan et al. [63] reported that, under iron limitation, the ArrF sRNA acts as a positive post-transcriptional regulator of the *phbR* gene. In the proposed mechanism, the *phbR* mRNA forms an inhibitory hairpin around the Shine–Dalgarno (SD) sequence in the ribosomal binding site (RBS), thereby preventing initiation of translation. When the levels of ArrF rise, this antisense sRNA binds to a complementary target sequence within the 5′ UTR of the *phbR* mRNA [63,130,136,137] (Figure 3B). In strain UW136, this interaction releases the inhibitory hairpin structure in the mRNA, unblocking the SD and allowing translation, which in turn increases PHB production [63]) (Figure 3B). However, in strain KCTC 23243 (whose wild-type is able to synthetize only small PHB quantities), this interaction results in a downregulation of *phbR* gene expression and therefore less accumulation of PHB [137]. 

#### 2.2.3. Global Post-Transcriptional Regulatory Protein Crc as Main Target of sRNAs CrcZ and CrcY in *Pseudomonas putida*

Pseudomonads are gram-negative bacteria able to adapt to a broad range of habitats and environments based on their metabolic versatility [138,139]. The species *P. putida* has been established as an important model system attributable to its biotechnological applications, among which is the synthesis of the bioplastics mcl-PHAs [49,50,139,140]. It owns an extraordinary number of regulatory systems and coordinating gene expression programs that allow it to adapt to various growth conditions according to the availability of substrates used for assimilation [141,142]. To coordinate the expression of genes involved in the transport and metabolism of these substrates, the assimilation of many of them is subject to carbon catabolic repression (CCR) when there is another compound in the medium that allows the bacteria to grow more efficiently. In this way, a hierarchical and sequential assimilation of the carbon sources present is facilitated, using the most favorable ones first, thereby improving growth rates and fitness [49,61,93,95,133,134,143,144,145].

The catabolite repression control protein (Crc) plays a key role in the CCR process, impeding the expression of genes involved in the synthesis of catabolic enzymes for the use of non-preferred carbon sources in pseudomonads [93,146,147,148]. This global regulator recognizes AANAANAA sequences in the genome called catabolite activity (CA) motif, located near the Shine–Dalgarno sequence of target mRNAs, and with a function in translation inhibition [61,91,93,143,148,149]. This process is in cooperation with the distal face of the protein Hfq, which is required by Crc to bind the mRNA motif through the formation of a stable ribonucleoprotein complex at the targets [95,96,149,150,151]. In Figure 9, Moreno et al. [93] summarizes the procedure by which the action of this complex is modulated and antagonized by two small RNAs (CrcZ and CrcY) in *P. putida.* The levels of both sRNAs significantly increase when bacteria grow with a non-preferred carbon source or have reached stationary growth phase. These sRNAs sequester one or both of the Crc/Hfq proteins, therefore decreasing the CCR, and allowing translation of the target mRNAs with A-rich motifs involved in the transport and/or assimilation of compounds [75,93,94,95,96,149,150,151]. During the formation of this multilayered and complex Hfq/Crc/CrcZ-CrcY regulatory system, the Crc–Hfq complex protects the sRNAs from ribonucleases by increasing their stability [53,96,101]. These sRNAs are mainly transcribed from σ54/RpoN-dependent promoters (*PcrcZ* and *PcrcY*) regulated by the two-component sensor-regulator system CbrA–CbrB (mainly CrcZ) together with other protein factors [61,75,93,94,95,96,148]. In this regulatory complex, each component affects either the transcription or the stability of the other components, e.g., the activity of the sRNA promoters relies on the type of carbon source and carbon/nitrogen (C/N) ratio. In turn, this promotes that the cellular metabolism adopts distinct pathways that allow the cell to adapt its requirements for energy and molecular biosynthesis [61,93,94,96,148]. 

Since PHAs are carbon and energy reserve sources, their continuous cycle of synthesis and degradation is expected to be affected by the carbon catabolic repression system [61]. In *P. putida* KT2440, the genes *phaC1* and *phaC2* encode for two PHA polymerases that incorporate (R)-3-hydroxyacyl-Coenzyme A, monomers derived from the beta-oxidation of fatty acids or via *de novo* synthesis, into the PHA polymer [152]. The hydrolysis of PHAs is carried out by the depolymerase encoded by the *phaZ* gene [25,49]. Other genes involved in the synthesis/degradation cycles are *phaF*, *phaI*, and *phaD*, which are responsible for the synthesis of phasin, structural proteins, and transcriptional regulators, respectively [25,49]. In *phaC1*, *phaF*, and *phaI* genes, sequences resembling CA motifs were found; however, the regulatory complex only inhibited the translation of *phaC1* polymerase mRNA, thus reducing the quantity of PHA synthesized in the cell. Neither the expression of *phaF* nor *phaI* was affected by the regulator. In line with this, the inhibitory action of Crc was not observed when the cultures entered into the stationary phase in media containing an unbalanced C/N ratio, especially with octanoic acid as a C source, in which PHA accumulation reached its maximum due to the antagonism of the sRNAs CrcZ and CrcY [25,49,61,153].

#### 2.2.4. Post-Transcriptional Control of *phaC1* Synthase as a Key Aspect along PHA Synthesis in *P. putida* CA-3

As mentioned above, the Gac–Rsm cascade is widely present in bacterial species and typically involves management of carbon storage, among others regulatory functions. Figure 3 of Ryan et al. [62] exposes the procedure whereby, in *Pseudomonas* species, a phosphor-transfer event would initiate the associated response of GacA of this two-component system by activating the transcription of the *rsmX*, *Y*, and *Z* sRNA genes, which in turn sequester the post-transcriptional regulator RsmA, allowing the mRNA translation and subsequent protein synthesis in a great variety of metabolic pathways [12,62]. 

To further analyze the role of this two component system in *Pseudomonas putida* CA-3, Ryan et al. [62] performed a screening of a random mini-Tn5 mutagenesis of its genome, in which 44 mutants were identified with a reduced PHA accumulation phenotype. After the characterization of one of these mutants (PHA45A) that has a disruption of the *gacS* gene, it was ultimately concluded that this sensor kinase is directly related with the post-transcriptional regulation of PHA synthesis in this strain [62,130]. To reach this conclusion, first the identification of Gac–Rsm cascade gene homologues in *P. putida* CA-3 was accomplished, followed by the evaluation of their genetic expression in both wild-type and *gacS* mutant backgrounds, under PHA accumulation conditions. However, and in contradiction with the model of transcriptional regulation within the cascade known in other pseudomonads, the transcription of the sRNAs RsmY and RsmZ (previously identified in strain CA-3), was not affected in the PHA45A mutant. Similarly, the expression of the PHA biosynthetic genes *phaC1* polymerase and *phaG*-encoded ACP-CoA transacylase [154], presented similar transcript levels in both genetic backgrounds, analyzed under the same conditions [62,130].

Despite these results in the expression of the analyzed genes, evidence exists that the *gacS* disruption in the PHA45A mutant of *P. putida* CA-3 inhibits PHA accumulation. Therefore, the possible regulation of the PHA synthesis at a post-transcriptional level was investigated [62]. Subsequently, the protein profile of the *gacS* mutant was evaluated together with an already characterized *phaC1*-disrupted mutant, whose protein had been previously reported to be essential for PHA accumulation in this strain [154]. Both strains exhibited an absence of protein at the expected ∼62 kDa band, compared with the wild-type protein profile. Hence, Ryan et al. [62] concluded that the post-transcriptional regulation of the PhaC1 PHA synthase was the key step in the GacS regulatory cascade along PHA synthesis in *P. putida* CA-3. This unusual procedure could involve other regulatory elements controlling RsmY and RsmZ sRNAs for PHA synthesis in *P. putida* CA-3, which would need further research to be fully understood [12,62,130].

## 3. Conclusions and Perspectives

PHAs are polyesters synthesized and biodegraded by microorganisms, which are produced from large accessible renewable resources and have potential use for numerous applications. However, detailed understanding and subsequent optimization of their production and purification are still mandatory to reduce their production costs [10,35,41,44].

### 3.1. Role of Post-Transcriptional Regulation during the Native Synthesis of PHAs

Free-living bacteria often need to develop flexible and versatile metabolic and regulatory networks to adapt to fast fluctuations in nutrient availability. Therefore, the destiny of C aims to maximize bacterial fitness and safety [151]. Phylogenetic analysis of the ability of bacteria and archaea to synthesize PHAs has revealed extensive horizontal gene transfer events of the genes and corresponding transcriptional regulators involved in the accumulation of these polymers [64,155]. However, in the vast majority of cases, their post-transcriptional regulation still remains unknown.

Riboregulation has a major role in the fine-tuning of multiple bacterial processes and is important to rapidly adjust cell growth in response to environmental changes [69]. sRNAs are non-translated small RNA molecules that are very important in the control of gene expression that usually silence their targets [68]. Ribonucleases are the enzymes that process and degrade all types of RNA and it is known that the RNA chaperone Hfq can protect RNA from the action of ribonucleases [53,81]. As shown in this review, the PHAs synthesis is also adjusted, directly or indirectly, through post-transcriptional regulation exerted by different kinds of RNAs molecules [61,62,63,64,128]. Although nothing has been published about the implication of RNases in the control of PHAs synthesis, they are expected to play an important role based on their marked importance in controlling other regulators and processes [54,72,156]. 

### 3.2. Controlling PHAs Production in Bacteria via Synthetic Small Non-Coding RNAs

**Figure 4:**
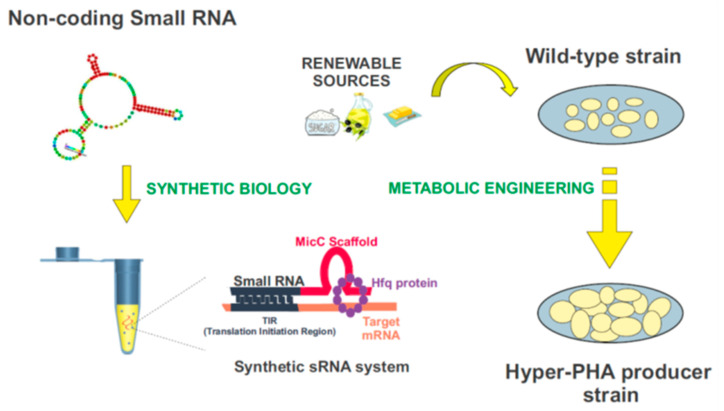
Biotechnological domestication of microorganisms to improve PHA synthesis. Synthetic biology and metabolic engineering strategies are used in the bacteria “domestication”. Customizable synthetic small RNAs could be rationally designed using the MicC scaffold to target genes of interest, and improve the industrial production of PHAs.

Synthetic biology is a compelling and expanding interdisciplinary research field which intends to provide a systematic framework for the design and construction of biological systems. It relies on the application of logical engineering principles to program or reprogram cellular functions at a genetic and metabolic level (Figure 4) [157]. One of the most important endeavors in contemporary synthetic biology is the search for optimal genomic chassis for industrial applications [158,159]. With this idea in mind, there has been a great effort to develop customizable regulators using genetic tools such as the CRISPR/Cas system, TALEs, and sRNAs [160], which would enable the precise control of gene expression, aiming to attain the desired functional outputs. Driven by the widespread role of post-transcriptional regulation in natural systems, the attention paid to RNA regulators is increasing [161]. Recent advances in nucleic acid engineering encourage the design of RNA components as building blocks in the construction of synthetic biological systems, mainly due to the plasticity of these molecules to interact with a myriad of proteins, metabolites, and other nucleic acids [162]. Synthetic RNA regulators display a wide range of programmable functions, offering important advantages over other protein-based mechanisms [156]. Among them, synthetic small non-coding RNAs (synthetic sRNAs) emerge as promising components to fine-tune gene expression. These customizable RNA regulators can be rationally designed to target different mRNAs, modulating their expression by altering their target-binding sequences (Figure 4) [12,156,163,164,165].

Improving the quality and reducing the costs in industrial production of PHAs is a matter of pressing importance. In the future, synthetic sRNAs could be used to domesticate bacteria throughout the modulation of their genetic expression, in particular on the enzymes involved in the PHAs synthesis. The construction of these customized sRNA systems could be used for this purpose, in combination with the use and further development of plasmid genetic tools, such as the SEVA plasmids, for the modulation of genes-of-interest [12,156,163,164,165,166,167]. Figure 4 exemplifies this process. The MicC scaffold can be used to design tailor-made sRNAs that target the genes of interest, with the help of the Hfq protein. More details about this synthetic sRNA system can be found in [156,163,164,165].

It is important to continue investing the biotechnological domestication of microorganisms using synthetic biology and metabolic engineering to implement the portfolio of PHAs and improve strategies to lower the costs in industrial production [12,168]. In this review, we have indicated many examples of how post-transcriptional control can be an instrumental tool for the regulation of polyhydroxyalkanoates synthesis.
